# Loss of TET2 increases B-1 cell number and IgM production while limiting CDR3 diversity

**DOI:** 10.3389/fimmu.2024.1380641

**Published:** 2024-03-27

**Authors:** Emily Dennis, Maria Murach, Cassidy M.R. Blackburn, Melissa Marshall, Katherine Root, Tanyaporn Pattarabanjird, Justine Deroissart, Loren D. Erickson, Christoph J. Binder, Stefan Bekiranov, Coleen A. McNamara

**Affiliations:** ^1^ Beirne B. Carter Center for Immunology Research, University of Virginia, Charlottesville, VA, United States; ^2^ Department of Microbiology, Immunology, and Cancer Biology, University of Virginia, Charlottesville, VA, United States; ^3^ Department of Biochemistry and Molecular Genetics, University of Virginia, Charlottesville, VA, United States; ^4^ Department for Laboratory Medicine, Medical University of Vienna, Vienna, Austria; ^5^ Division of Cardiovascular Medicine, Department of Medicine, University of Virginia, Charlottesville, VA, United States

**Keywords:** innate B cells, B-1 cells, ten-eleven translocation-2 (TET2), natural antibodies (Nab), immunoglobulin M (IgM), B cell receptor (BCR), complementarity-determining region-3 (CDR3)

## Abstract

Recent studies have demonstrated a role for Ten-Eleven Translocation-2 (TET2), an epigenetic modulator, in regulating germinal center formation and plasma cell differentiation in B-2 cells, yet the role of TET2 in regulating B-1 cells is largely unknown. Here, B-1 cell subset numbers, IgM production, and gene expression were analyzed in mice with global knockout of TET2 compared to wildtype (WT) controls. Results revealed that TET2-KO mice had elevated numbers of B-1a and B-1b cells in their primary niche, the peritoneal cavity, as well as in the bone marrow (B-1a) and spleen (B-1b). Consistent with this finding, circulating IgM, but not IgG, was elevated in TET2-KO mice compared to WT. Analysis of bulk RNASeq of sort purified peritoneal B-1a and B-1b cells revealed reduced expression of heavy and light chain immunoglobulin genes, predominantly in B-1a cells from TET2-KO mice compared to WT controls. As expected, the expression of IgM transcripts was the most abundant isotype in B-1 cells. Yet, only in B-1a cells there was a significant increase in the proportion of IgM transcripts in TET2-KO mice compared to WT. Analysis of the CDR3 of the BCR revealed an increased abundance of replicated CDR3 sequences in B-1 cells from TET2-KO mice, which was more clearly pronounced in B-1a compared to B-1b cells. V-D-J usage and circos plot analysis of V-J combinations showed enhanced usage of V_H_11 and V_H_12 pairings. Taken together, our study is the first to demonstrate that global loss of TET2 increases B-1 cell number and IgM production and reduces CDR3 diversity, which could impact many biological processes and disease states that are regulated by IgM.

## Introduction

B cells participate in both innate and adaptive immunity through the secretion of antibodies. B cells are broadly divided into B-1 and B-2 subtypes. B-2 cells are derived from hematopoietic progenitor cells in the bone marrow (BM) and function predominantly in T cell-dependent responses for antibody production (1, 2). B-1 cells originate during early fetal life, are long-lived, and self-renew (3–6). B-1 cells can be found predominantly in serosal spaces such as the peritoneal cavity (PerC) or the pleural cavity but can also be found in secondary lymphoid organs such as the spleen, lymph nodes, and the BM (7). B-1 cells are further subtyped into B-1a or B-1b cells depending on the expression of CD5 (B-1a are CD5+). B-1 cells produce about 80% of circulating serum IgM (immunoglobulin M). A low level of IgM is produced by B-1 cells in serosal cavities, and the majority of circulating serum IgM is produced by B-1 cells in the spleen and BM (8–10). IgM antibodies produced by B-1a cells are thought to be naturally occurring (i.e., present at birth, in gnotobiotic mice and without antigen exposure) (11–13). These natural antibodies provide rapid protection from infections and maintain tissue homeostasis through apoptotic cell clearance (10, 14). However, recent evidence identified the VDJ region in B-1a cells as having N additions (3, 15–18), an event due to the action of the DNA polymerase TdT which is only expressed after birth. This suggests more complexity to the regulation of the CDR3 in B-1a cells than previously thought.

The TET family of proteins act enzymatically as α-ketoglutarate-dependent cytosine dioxygenases that promote DNA demethylation by oxidizing the methyl group of 5-methylcytosine (5mC) to 5-hydroxymethylcytosine (5hmC) (19–21). The methylation status of DNA is important in recruiting proteins for gene repression or inhibition of transcription factor binding. Additionally, TET proteins enlist chromatin-modifying proteins to histones, which can affect gene expression via physical accessibility for transcription (22, 23). Thus, TET proteins are potent epigenetic modulators. TET2 is involved in hematopoietic cell development and differentiation (24–26). Dysfunction in TET2 is well characterized in hematological malignancies including acute myeloid leukemia (AML) (27–30) and myelodysplastic syndrome (MDS) (30–34). TET2 loss can affect inflammatory responses via altered cytokine secretion (35, 36) and other biological processes in myeloid cells (26, 30, 37–40). TET2 has also been implicated in B cell lymphomas (22, 41–47). Most studies of TET2 in B cells primarily focused on B-2 cells and suggested reduced production of high-affinity IgG (42–44). Only one study to date has briefly investigated TET2 loss in B-1 cells, and that was with a focus on diffuse large B cell lymphoma and chronic lymphocytic leukemia development (46). In contrast, our study focuses on B-1 cells in young mice without evidence of tumor, allowing for the identification of key homeostatic processes that may be altered by loss of TET2. Our novel findings characterize the impact of global loss of TET2 on B-1 cell biology at homeostasis, revealing that global TET2 loss leads to increased B-1 cell number, IgM production, and the number of replicated complementarity-determining region 3 (CDR3) sequences, which could impact diseases that are modulated by IgM antibodies to specific antigens.

## Materials and methods

### Mice

All animal protocols were approved by the Animal Care and Use Committee at the University of Virginia. TET2-KO mice (24) were provided by Dr. Kenneth Walsh (University of Virginia). The model was generated by Ko et al. and targeted the endogenous *TET2* locus to create a conditional allele that enabled the deletion of exons 8, 9, and 10, the catalytic region of TET2 (24). Mice were maintained on a 12-h light/dark schedule in a specific pathogen-free animal facility and given food (standard chow diet, Tekland 7012) and water *ad libitum*. The number of mice included in each study is indicated in the figures or the associated legends.

### Sample preparations for flow cytometry and live cell sorting

Bone marrow, spleen, and peritoneal cavity cells were processed for flow cytometry as previously described (48). Briefly, following sacrifice by CO_2_ overdose, peritoneal cells were harvested by flushing the peritoneal cavity with 10 mL FACS buffer (PBS containing 1% BSA, 0.05% NaN3). The spleen and one femur and tibia were removed. Spleens and flushed bone marrow were filtered through a 70 μm cell strainer. Red blood cells were lysed from single-cell suspensions of bone marrow and spleen using a lysis buffer containing 155 mM NH_4_Cl, 10 mM KHCO_3_, and 0.1 mM EDTA. Cell surface Fc receptors were blocked using anti-CD16/32 (clone:93, 4 eBioscience), then cells were stained with fluorescently conjugated antibodies against cell surface markers. Cells were stained with fixable Live/Dead Zombie NIR (Life Technologies) for dead cell discrimination, then fixed in 2% PFA in PBS. For FAC sorting, cells were resuspended in modified FACS buffer (PBS with 1% BSA) and 4’,6-Diamidino-2-Phenylindole (DAPI) live/dead stain then immediately taken to the University of Virginia Flow Cytometry Core for sorting. B-1a and B-1b cells were sorted to better than 99% purity from their parent gate. Clone and fluorophore information for the flow cytometry antibodies used in murine experiments to immunophenotype or FAC-sort B cell subsets are given in **Tables 1**, **2** respectively. All flow cytometry was conducted at the University of Virginia Flow Cytometry Core Facility. Immunophenotyping was performed on an Aurora Borealis 5-laser (Cytek) cytometer. FAC-sorting was performed on an Influx Cell Sorter (Becton Dickinson). Data analysis and flow plots were generated using OMIQ software (Dotmatics). Representative flow plots were chosen based on the samples whose population frequencies were closest to the mean for that group. Gates on flow plots were set using fluorescence minus one (FMO) controls.

**Table 1:** The immunophenotyping panel used for flow cytometry results presented in **Figure 1**.

Marker	Fluorophore	Clone	Vendor
CD45	PerCP	30-F11	BD
B220	APC	RA3-6B2	eBioscience
CD19	APCefl780	1D3	eBioscience
IgM	PECF594	R6-60.2	BD
IgD	efl450	11-26	eBioscience
CD8	BV510	53-6.7	BioLegend
CD4	PECy5.5	GK1.5	SouthernBiotech
CD44	BV785	IM7	BioLegend
CD62L	BV570	MEL-14	BioLegend
CD25	BB515	PC61	BD
F4/80	PECy7	BM8	eBioscience
CD11b	PerCPCy5.5	M1/70	BD
CD11c	AF647	N418	BioLegend
CD138	PE	281-2	BD
Ly6c	BV711	HK1.4	BioLegend
NK1.1	BV480	PK136	BD
CD5	BV605	53-7.3	BD
CD21	FITC	4E3	eBioscience
CD23	BUV737	B3B4	BD
Zombie NIR Fixable Viability Dye			BioLegend

**Figure 1:**
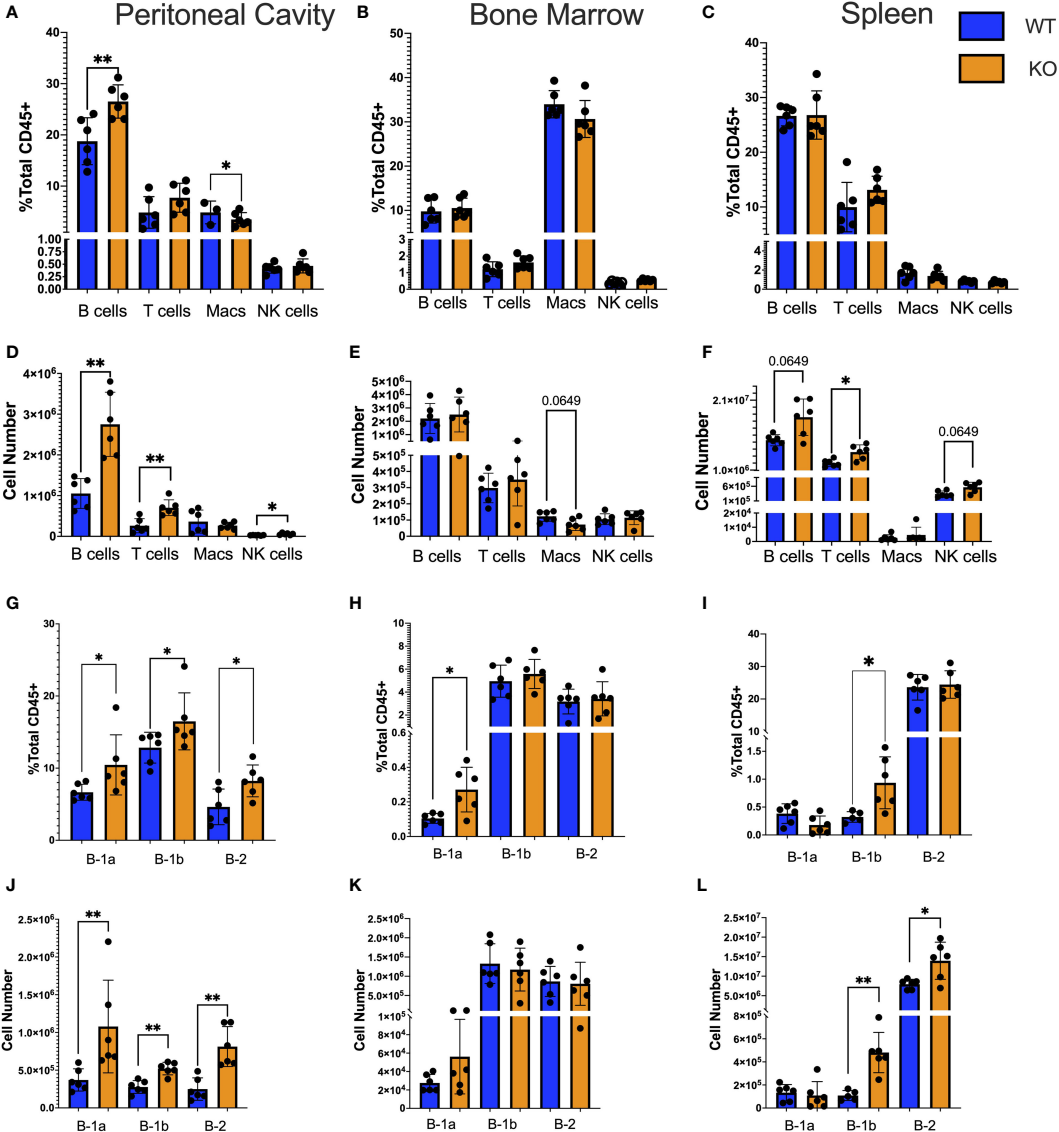
Immune subtypes in TET2-KO compared to WT mice. **(A-L)** Flow cytometry characterization of the number and frequency of immune cell types in the **(A, D)** peritoneal cavity; **(B, E)** bone marrow; **(C, F)** spleen. Deeper gating into B cell subset frequency and number in the **(G, J)** peritoneal cavity; **(H, K)** bone marrow; **(I, L)** spleen, respectively, from TET2-KO (n = 6) and WT (n = 6) mice. Blue and orange represent WT and TET2-KO mice, respectively. Data are representative of four independent experiments. Significance was determined with two-tailed Mann-Whitney U-tests (*p < 0.05, **p < 0.01).

**Table 2:** The FACS panel used for sorting B cell subsets from the peritoneal cavity presented in **Figure 2**.

Marker	Fluorophore	Clone	Vendor
IgD	FITC	11-26	eBioscience
CD5	PE	53-7.3	eBioscience
CD23	PE-CY7	B3B4	eBioscience
B220	APC	RA3-6B2	eBioscience
CD19	APC-EF780	1D3	eBioscience
DAPI Staining Solution			Miltenyi Biotec

**Figure 2:**
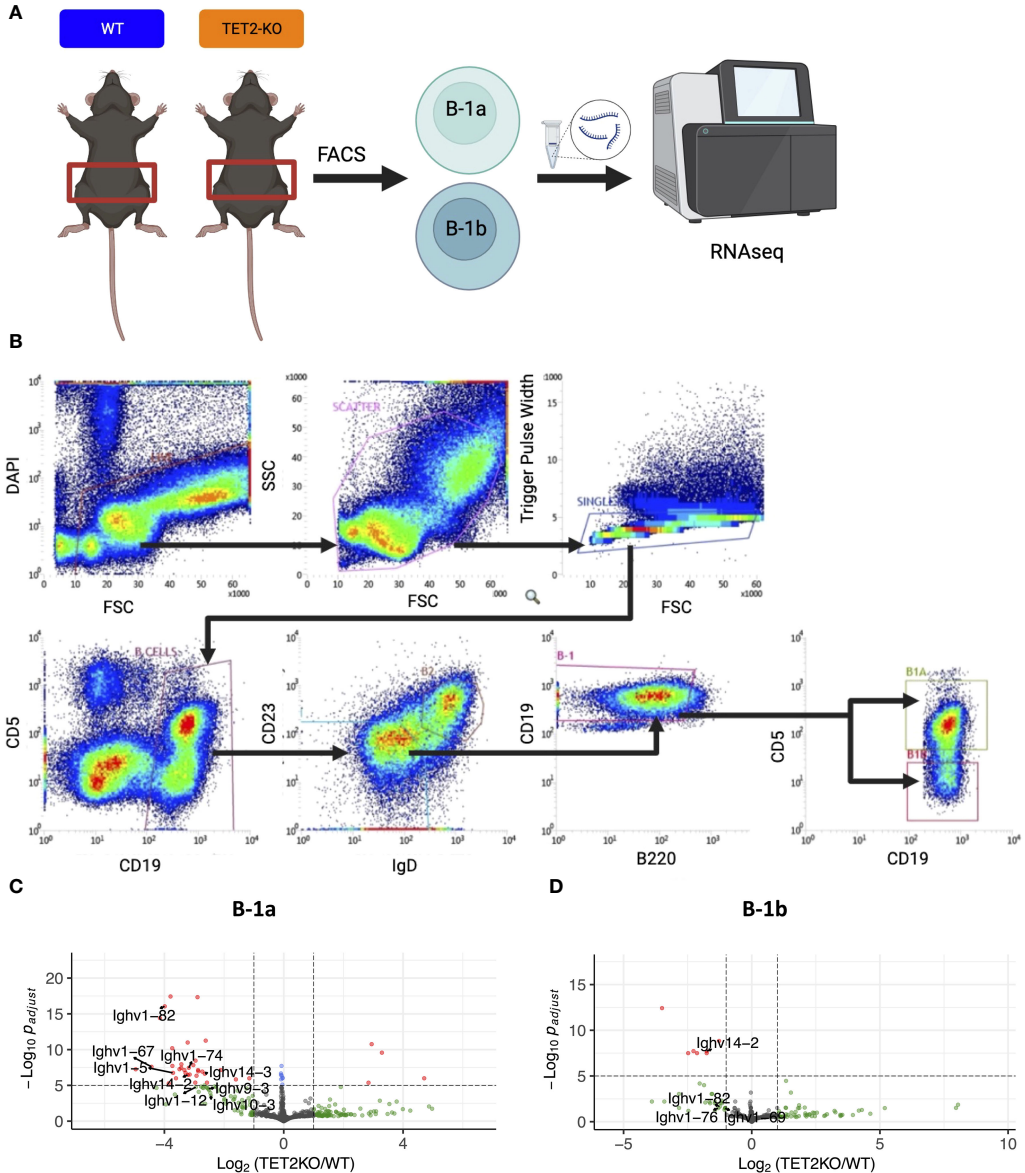
RNASeq analysis of differentially expressed genes in peritoneal B-1a and B-1b cells from TET2-KO and WT mice. **(A)** Schematic of experimental design. B-1a and B-1b cells from the peritoneal cavity of TET2-KO and WT mice were sort-purified and RNA-extracted for RNASeq. **(B)** Gating strategy for sort. B-1a cells are CD19+, IgD-lo, CD23-lo, B220-lo, CD5+ while B-1b cells are CD19+, IgD-lo, CD23-lo, B220-lo, CD5-. **(C, D)** Differentially expressed genes are visualized with volcano plots of the B-1a **(C)** and B-1b **(D)** cells from TET2-KO mice compared to WT. Color legend for volcano plots: Grey – NS, Green: log2FC > 1, Blue: p-value < 0.05 and log2FC < 1, Red: p-value < 0.05 and log2FC > 1. n: B-1a: WT = 4, KO = 4, B-1b: WT = 4, KO = 3. All p-values are False Discovery Rate (FDR)-adjusted. Figure schematic made with BioRender.

### ELISA for quantification of total IgM in mice

Total IgM in mouse plasma was measured using colorimetric ELISA as described previously (48). Briefly, EIA/RIA high-binding microplates were coated with goat anti-mouse IgM, capture antibody (Southern Biotech, 1020-01). Mouse IgM standards (Southern Biotech, 0101-01), or plasma samples were detected with alkaline phosphatase-conjugated goat anti-mouse IgM secondary antibody (Southern Biotech, 1020-04); and pNPP substrate (Southern Biotech 0201-01). Absorbance measurements were analyzed with a SpectraMAX 190 microplate reader (Molecular Devices) at 405 nm. The standard curve was determined using a 4-parameter function and concentration measurements were extrapolated using Softmax Pro 3.1.2 software. Only samples with CV<15% and within the standard curve were included in the analysis.

### Sample preparation for bulk RNA sequencing

Peritoneal B-1a, B-1b, and B-2 cells obtained from *TET2-*KO and *TET2-*WT C57BL/6 mice were sort-purified directly into RLT Plus Buffer (Qiagen). RNA and DNA were extracted using the Qiagen AllPrep kit. The purified RNAs were stored at −80°C before being sent to Novogene for sequencing. Total RNA was stored in RNase-free water to directly synthesize first strand, followed by the whole-length LD-PCR amplification. The amplified ds-cDNA(double-stranded DNA) was purified with AMPure XP beads and quantified with Qubit. The cDNA samples were sheared by the Covaris system, and then the sheared fragments were end-repaired, A-tailed, and ligated to sequencing adaptors. A size selection of about 200 bp was performed before the PCR enrichment. Library concentration was first quantified using a Qubit 2.0 fluorometer (Life Technologies), and then diluted to 2 ng/µl before checking insert size on an Agilent 2100 and quantifying to greater accuracy by qPCR. Ultra-low input bulk RNA sequencing was performed on the NovaSeq 6000 PE150 (Illumina).

### DEG and pathway analysis

The quality trimming was performed using fastp (49) with default settings. Mapping to the GRCm39 genome was performed with STAR (50), followed by featureCounts (51) to count reads mapped to genes. DESeq2 (52) was used for differential analysis, followed by pathway analysis using clusterProfiler (53) with the Gene Ontology (GO) (54) database.

### BCR analysis

For BCR analysis, quality trimming was performed using fastp (49) and TRUST4 (55) was subsequently used to identify BCR repertoire in paired sequencing reads using the international ImMunoGeneTics (IMGT) information system database as a reference. Results were analyzed using R and circus plots were made using circos Bioconductor package (56). The code developed for these analyses will be available on the following Github page: https://github.com/mariamurach/TET2 and https://github.com/mariamurach/bcr_R.

### Statistics

**Figure 3:**
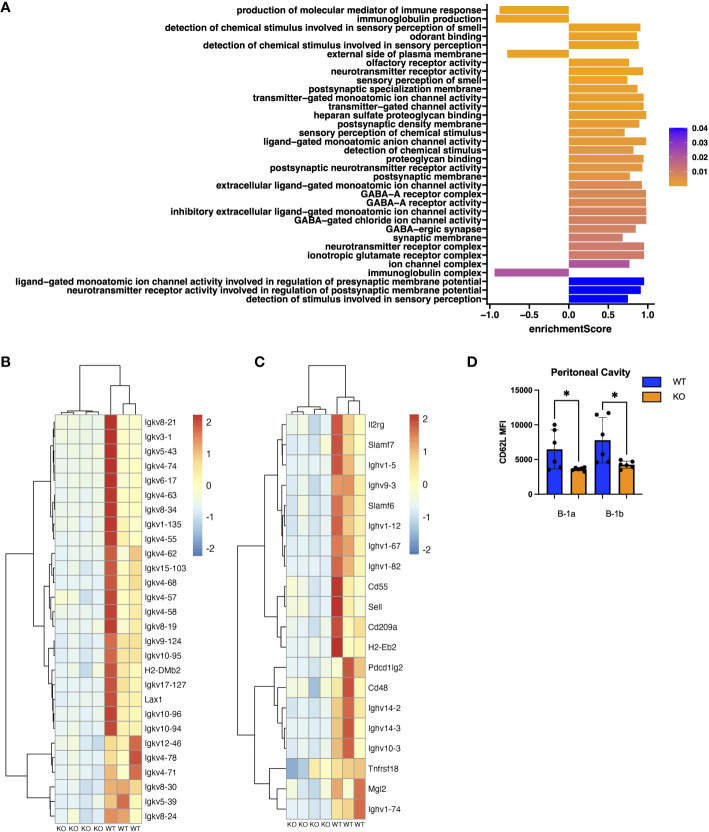
Pathway and Gene Set Enrichment Analysis (GSEA) of peritoneal B-1a cells from TET2-KO mice. **(A)** Plot of enrichment scores from GSEA on differentially expressed genes in B-1a cells from TET2-KO and WT mice. The axis represents the enrichment score (ES). Higher scores indicate greater enrichment of the gene set at one end of the ranked list of genes. ES measure the degree to which a gene set is overrepresented at the extremes of the entire ranked list. ES are colored based on FDR-adjusted p-values. **(B)** Scaled expression of genes involved in the production of molecular mediators of immune response and immunoglobulin production pathways. Each row corresponds to a gene, and each column represents a WT or TET2-KO sample. The expression was scaled for each gene (from -2 to 2) and is represented by the color red for high and blue for low expression values. **(C)** Scaled expression of genes differentially expressed and found on the cell surface. Each row corresponds to a gene, and each column represents a WT or TET2-KO sample. The expression was scaled for each gene (from -2 to 2) and is represented by the color red for high and blue for low expression values. **(D)** Bar chart displaying Median Fluorescence Intensity (MFI) of CD62L (Sell) in peritoneal B-1a cells and B-1b cells from TET2-KO and WT mice. Blue and orange represent WT (n = 6) and TET2-KO mice (n = 6), respectively. Significance was determined with two-tailed Mann-Whitney U-tests (*p < 0.05). n: B-1a: WT = 4, KO = 4 for **(A-C)**.

**Figure 4:**
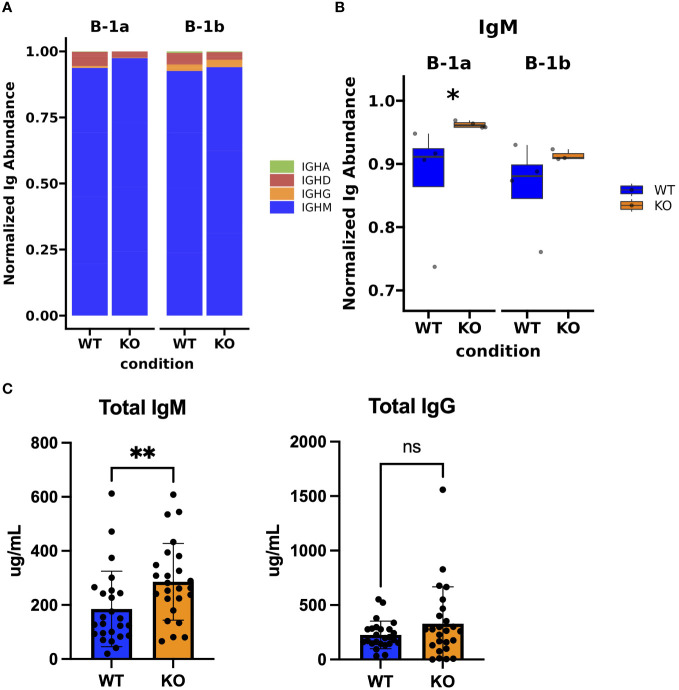
Immunoglobulin isotype analysis in peritoneal B-1a and B-1b cells from TET2-KO and WT mice. **(A)** Bar chart showing the distribution of Ig isotypes identified by TRUST4 in B-1a and B-1b cells from TET2-KO and WT mice. **(B)** Proportion of IgM expression in B-1a and B-1b cells from TET2-KO and WT mice. **(C)** Enzyme-linked immunosorbent assay (ELISA) of total IgM (left) and IgG (right) from plasma of TET2-KO (n = 26) and WT (n = 26) mice. Blue and orange represent WT and TET2-KO mice, respectively. Significance was calculated using Wilcoxon Rank Sum (*p < 0.05, **p < 0.01) for panel **(B)** Significance was determined with two-tailed Mann-Whitney U-tests (*p < 0.05, **p < 0.01, ns, not significant) for **(C)** n: B-1a: WT = 4, KO = 4, B-1b: WT = 4, KO = 3 in **(A, B)**.

**Figure 5:**
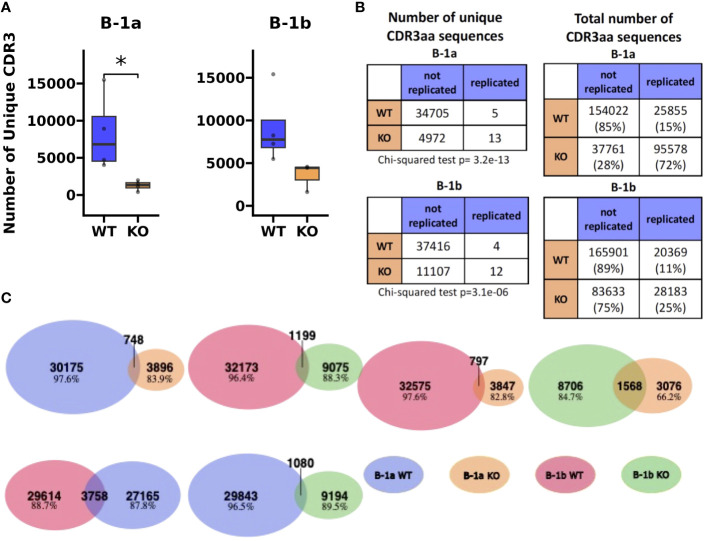
Heavy chain CDR3 sequence analysis reveals restricted BCR repertoire in peritoneal B-1a and B-1b cells from TET2-KO and WT mice. **(A)** The number of unique CDR3 sequences identified by TRUST4 in B-1a and B-1b cells from TET2-KO and WT mice. **(B)** Contingency tables derived to assess the association between the number of unique CDR3 amino acid sequences (left) and total number of CDR3 amino acid sequences (right) with the mutant status of the mice (i.e., WT or TET2-KO) in B-1a (top) and B-1b cells (bottom). Chi-squared test was used to assess the significance of these associations. Significance in **(A)** was calculated using Wilcoxon Rank Sum (*p < 0.05). **(C)** Venn diagrams that examine the shared repertoire of unique CDR3 sequences in the different B cell subsets. Blue represents CDR3 AA sequences from B-1a cells from WT mice, orange from B-1a cells from TET2-KO mice, red B-1b cells from WT mice, and green B-1b cells from TET2-KO mice, respectively. A shared sequence was defined as one expressed at least once in each of the subsets being compared. The number of shared sequences is represented by the overlapping region in each Venn diagram. Numbers and percentages of nonshared sequences of each cell subset in every comparison are indicated. For **(B, C)**, sequences were pooled from mice from the same cell type and condition (n: B-1a: WT = 4, KO = 4, B-1b: WT = 4, KO = 3).

In **Figures 1**, **3D**, **4C**, comparisons were conducted between the TET2-KO and WT strains using Prism 10.0 with unpaired, two-tailed Mann-Whitney U-tests. Values shown are mean ± SD. In **Figures 3B**, **4A** Wilcoxon Rank Sum and Signed Rank Tests were used to determine the significance of differences in proportions of unique CDR3 sequences, isotypes, and usages of specific V, D, and J chains between TET2-KO and WT groups. In **Figure 5B**, chi-squared test was performed to assess the significance of the association between the number of unique CDR3 amino acid sequences in B-1a and B-1b cells from TET2-KO and WT mice.

## Results

### Global loss of TET2 results in increased numbers of all B cell subtypes in the peritoneal cavity compared to WT

To determine the impact of the loss of TET2 on major immune cell subtypes in the peritoneal cavity, BM, and spleen of TET2-KO and littermate control mice, spectral flow cytometry was performed (**Figure 1**). B cells were defined as CD45+ CD19+; T cells were defined as CD45+ CD5+ CD19-; Macrophages (Macs) were defined as CD45+ CD5- CD19- F4-80+ CD11b+; and NK cells were defined as CD45+ CD5- CD19- NK1.1+ (**Supplementary Figure 1**). We found that there was a higher B cell frequency and number in the peritoneal cavity (**Figures 1A, D**), but not in the BM (**Figures 1B, E**) or spleen (**Figures 1C, F**) of TET2-KO mice compared to controls. Numbers of T cells (p-value = 0.0043), and NK cells (p-value = 0.0152) from TET2-KO mice in the peritoneal cavity (**Figure 1D**) were also greater than controls. There were no significant differences in immune cell numbers from TET2-KO mice in the bone marrow (**Figure 1E**), while in the spleen there was a trending increase in B cells (p-value = 0.0649) with a significant increase in T cells (p-value = 0.0260) and a trending increase in NK cells (p-value = 0.0649) compared to WT (**Figure 1F**). Upon examination of B cell subsets specifically, we found that in the peritoneal cavity, all B cell subsets were significantly increased in frequency (B-1a p-value = 0.0152, B-1b p-value = 0.0411, B-2 p-value = 0.0260) and in number (B-1a p-value = 0.0022, B-1b p-value = 0.0043, B-2 p-value = 0.0022) in TET2-KO mice compared to WT (**Figures 1G, J**). However, in the BM (**Figures 1H, K**) only the B-1a cell subset frequency was significantly increased in TET2-KO compared to WT mice (p-value = 0.0260). In the spleen (**Figures 1I, L**) B-1b cells but not B-1a cells were elevated in both frequency and number (B-1a p-value = 0.0173, B-1b p-value = 0.0043). There was no difference in TET2-KO B-2 cell frequency in the spleen, although the total number of B-2 cells was significantly increased (p-value = 0.0260).

### Peritoneal B-1a cells from TET2-KO mice have lower expression of immunoglobulin genes compared to WT

To identify genes differentially expressed in B cell subtypes in mice with TET2-KO compared to WT control, we performed RNA-sequencing (RNASeq) on sort-purified peritoneal B-1a and B-1b cells from TET2-KO and WT mice (**Figures 2A, B**; **Supplementary Figure 2**). We utilized peritoneal B-1 cells due to their abundance in this specific niche, as well as due to the phenotypic changes we observed in **Figure 1**. We found that the global knockout of TET2 had a more significant impact on gene expression within B-1a cells compared to B-1b cells. Specifically, we observed a downregulation in the expression of several immunoglobulin genes in B-1a cells (and to a lesser extent in B-1b cells) from TET2-KO mice compared to their WT counterparts (**Figures 2C, D**; **Supplementary Figure 3**). Consistent with this finding, Gene Set Enrichment Analysis (GSEA) revealed that TET2 loss markedly affects pathways linked to immunoglobulin production and immune response activation, primarily within B-1a cells (**Figure 3A**). Indeed, the expression of numerous V genes (from both heavy and light chains) was decreased in B-1a cells from TET2-KO mice (**Figures 3B, C**). Similarly, the expression of genes involved in the activation of molecular mediators of the immune response was also decreased in these cells (**Supplementary Figures 3, 5**). Interestingly, significant enrichment in neurotransmitter and synapse-related pathways was seen in both B-1a and B-1b cells from the TET2-KO animals compared to the control animals (**Supplementary Figure 3B**, These GSEA results provide potential avenues for further hypothesis-driven studies of the role of TET2 in sensory-neural control of B cells, an emerging area of potential significance, recently also connected to cardiovascular disease development via other immune cells (57–59). Notably, while there are too many differentially expressed genes (DEGs) to test all at the protein level, one of the proteins encoded by our DEG, Sell, also known as CD62L, was also in our flow panel, allowing us to determine if the change in gene expression was also accompanied by changes in the protein level. Indeed, consistent with the decrease in CD62L RNA, we also saw a decrease in CD62L on the surface B-1a and B-1b cells in TET2-KO mice (**Figures 3C, D**).

### Global loss of TET2 results in higher expression of IgM antibody isotype in peritoneal B-1a cells compared to WT

Using TRUST4, a tool for analyzing the B cell receptor (BCR) using bulk RNASeq (57) and the IMGT (58) database, we were able to identify Ig isotype transcripts present in sequencing data and their distribution across B-1a and B-1b cells from TET2-KO and WT mice (**Figure 4A**). We found that there was a statistically significant increase in the expression of IgM in the B-1a cells from TET2-KO mice, but we do not see that effect in B1-b cells which is in accordance with the increase expression of *AIDCA*, a gene involved in class-switch recombination (**Figure 4B**; **Supplementary Figure 5**). In contrast, there was not a significant change in the distribution of IgD, IgG, or IgA isotypes expressed by the different B-1 cells in TET2-KO and WT mice (**Figure 4A**). Consistent with the increase in B-1 cells that we observed in niches that support antibody production, such as the spleen and BM (**Figures 1E, F**), and the increase in the IgM transcript in B-1a cells, circulating plasma IgM levels were higher in the TET2-KO compared to WT mice (p-value = 0.0075) (**Figure 4C**). Marginal zone B-2 cells (MZB) are another source of IgM and we did observe an increase in MZB cell number in TET2-KO mice compared to controls, which could contribute to the overall increase in circulating IgM (**Supplementary Figure 6**). We observed no change in circulating IgG levels.

### Global loss of TET2 results in a reduced number of unique heavy chain CDR3 sequences and an increased number of replicated heavy chain CDR3 sequences in peritoneal cavity B-1a cells compared to WT

**Figure 6:**
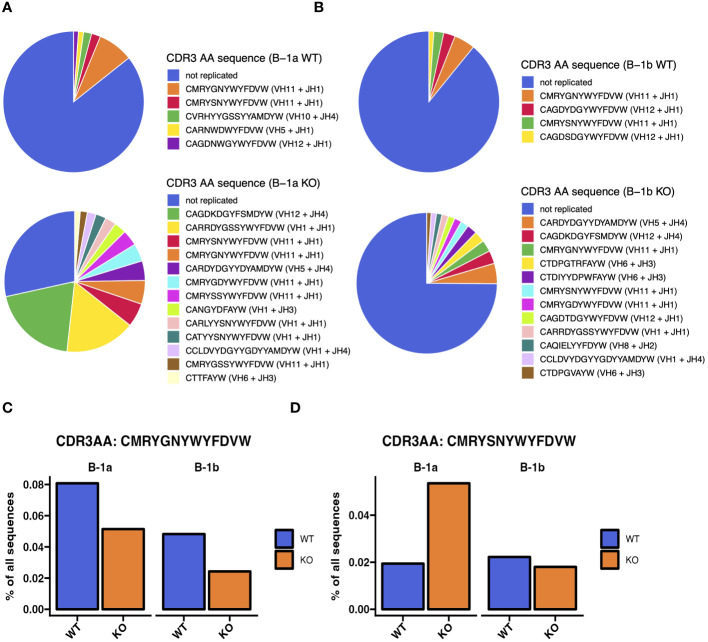
Heavy chain CDR3 sequence analysis reveals differences in most abundant CDR3 sequences in peritoneal B-1a and B-1b cells from TET2-KO and WT mice. **(A, B)** Annotated pie charts depicting the proportion of CDR3 sequences that are unique and the sequence and proportion of the replicated sequences in B-1a **(A)** and B-1b **(B)** cells from WT (top) and TET2-KO mice (bottom). **(C)** Bar chart comparing the proportion of the top-most abundant CDR3 sequence in B-1a cells from WT mice from of all CDR3 sequences in B-1a and B-1b cells from TET2-KO and WT mice. **(D)** Bar chart comparing the proportion of the second-most abundant CDR3 sequence in B-1a cells from WT mice from of all CDR3 sequences in B-1a and B-1b cells from TET2-KO and WT mice. Blue and orange represent WT and TET2-KO mice, respectively. Sequences were pooled from mice from the same cell type and condition (n: B-1a: WT = 4, KO = 4, B-1b: WT = 4, KO = 3).

To assess differences in the heavy chain BCR repertoire in B-1 cells from TET2-KO and WT mice, we performed an analysis of the CDR3 sequences using our bulk RNASeq data and TRUST4 (57). Results demonstrated that in both B-1a and B-1b cells from TET2-KO mice, CDR3 diversity was reduced compared to WT mice (**Figure 5A**). The reduction in CDR3 sequence diversity in the B-1a cells from TET2-KO mice compared to WT was statistically significant (p-value = 0.02857) (**Figure 5A**), while the reduction in unique CDR3 sequences in TET2-KO B-1b cells compared to WT was trending (p-value = 0.05714) (**Figure 5A**). It is not feasible to establish the presence of clonal expansion based on bulk RNASeq data, due to the inability to determine the absolute number of cells and their level of expression of each Ig transcript at a single-cell resolution in a given B cell population. However, a high proportion of replicated sequences suggests the presence of clonally dividing, or self-renewing B-1a cells, as they are known to do. Here we define replicated sequences as those whose frequency is greater than 1% of all sequences. In B-1a cells from TET2-KO mice, we observed that 72% of the CDR3 sequences were replicated, compared to B-1a cells from WT mice which only had 15% of the total CDR3 sequences replicated (**Figures 5B**, **6A**). Thirteen unique CDR3 sequences covered 72% of all CDR3s in B-1a cells from TET2-KO mice, while 4972 CDR3 sequences made up the other 28% of the total number of identified CDR3s (**Figures 5B**, **6A**). In B-1b cells from TET2-KO mice, 25% of all CDR3 sequences are made up of 12 unique CDR3 sequences, while 11107 CDR3 sequences made up the rest of the 75% (**Figures 5B**, **6B**). Differences in the number of replicated unique CDR3 sequences were significant based on Chi-squared tests for B-1a (p-value = 3.2x10^-13^) and B-1b cells (p-value = 3.1x10^-6^) from TET2-KO mice compared to WT (**Figure 5B**). An analysis of the commonality of replicated CDR3 sequences revealed that there was minimal overlap in the CDR3 sequence between B-1a cells from TET2-KO and WT mice or in B-1b cells from TET2-KO and WT mice, respectively (**Figure 5C**). Interestingly, there was a greater degree of commonality in the CDR3 sequence comparing B-1a and B-1b cells from TET2-KO mice. We visualized the proportion of replicated CDR3 sequences across B-1 cell subsets in TET2-KO and WT mice using pie charts (**Figures 6A, B**). Consistent with our findings in **Figure 5C**, the CDR3 sequences that were most abundantly represented in B-1a cells from WT mice were represented in B-1a cells from TET2-KO mice at different proportions, and there were more similarities in replicated CDR3 sequences between B-1a and B-1b cells from TET2-KO mice (**Figures 6C–D**).

Since antigen binding specificity is not just determined by the heavy chain CDR3, we performed an analysis of the light chain BCR repertoire from our bulk RNASeq data with TRUST4. Results demonstrated that in both B-1a and B-1b cells from TET2-KO mice, CDR3 diversity was reduced compared to WT mice (**Supplementary Figure 7**). The reduction in CDR3 sequence diversity in the B-1a cells from TET2-KO mice compared to WT was statistically significant (p-value = 0.029) (**Supplementary Figure 7A**). The reduction in CDR3 sequences in B-1b cells from TET2-KO mice compared to WT was trending (p-value = 0.133) (**Supplementary Figure 7A**). Similar to what we observed in the heavy chain CDR3 sequences, the number of replicated light chain CDR3 sequences was over 2-fold greater in B-1a cells from TET2-KO compared to WT mice (**Supplementary Figures 7B-D**). The number of unique CDR3 sequences from the light chain accounted for a similar percentage of total CDR3 sequences as seen in the heavy chain results in B-1a cells from TET2-KO mice (**Supplementary Figures 7B-D**). Differences in the number of replicated unique CDR3 sequences were significant based on Chi-squared tests for B-1a (p-value = 0.01) and B-1b cells (p-value = 0.02) from TET2-KO mice compared to WT. While the role of the light chain in antigen binding and specificity remains less well-known compared to the heavy chain, it still contributes to those functions (59). These results in the light chain CDR3 provide additional support that B-1a cells are more profoundly impacted by loss of TET2 than B-1b cells, and the diversity of antigen-specific IgMs may be affected as a result.

### V_H_–D_H_–J_H_ usage shows differences between TET2-KO and WT BCR repertoires

Analysis of specific V_H_–D_H_–J_H_ gene region usage in B-1a and B-1b cells from TET2-KO and WT mice revealed high usage of V_H_1, V_H_11, and V_H_12 in B-1 cells consistent with prior findings (**Supplementary Figure 8**) (18, 60, 61). The B-1a cells from the TET2-KO mouse appeared to have greater usage of these regions. There were also several reductions in V_H_ region usage in the B-1a cells from the TET2-KO mice, but these were regions of minimal usage and of unclear significance. We also analyzed differences in the specific V_K/L_–J_K/L_ gene regions of the light chain CDR3 sequence in B-1a and B-1b cells from TET2-KO or WT mice (**Supplementary Figure 9**) and similarly found differences in V_K_ and J_K_ usage predominantly in B-1a compared to B-1b cells. An analysis of kappa and lambda ratio revealed that there is more lambda light chain utilization in B-1b cells from TET2-KO mice compared to WT despite not reaching significance, while showing no difference in kappa/lambda ratio in B-1a cells (**Supplementary Figure 9E**).

**Figure 7:**
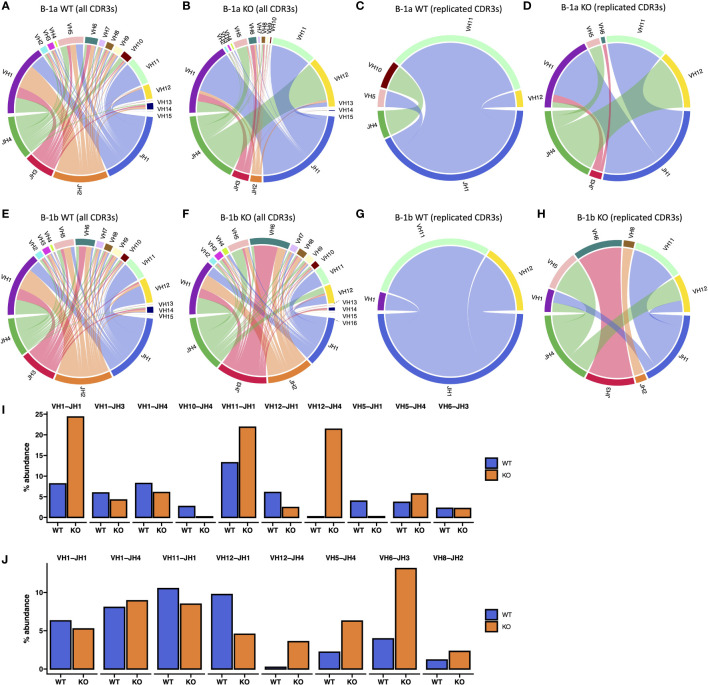
V-J Gene Association analysis of B-1a and B-1b cells from TET2-KO and WT mice. **(A-H)** Circos plot of V-J gene associations of CDR3 sequences identified by TRUST4 utilizing specific V-J gene segment pairs is displayed for B-1a cells from TET2-KO **(B, D)** and WT mice **(A, C)** from all CDR3 sequences **(A, B)** and replicated CDR3 sequences **(C, D)**. Circos plot of V-J gene associations of CDR3 sequences identified by TRUST4 utilizing specific V-J gene segment pairs is displayed for B-1b cells from TET2-KO **(F, H)** and WT mice **(E, G)** from all CDR3 sequences **(E, F)** and replicated CDR3 sequences **(G, H)**. Cables connect V and J gene segments that are observed together within the same CDR3 region, with the thickness of each cable indicating the relative frequency of each V-J pairing. **(I, J)** The abundance of V-J gene connections identified in replicated CDR3s presented as percent abundance of all CDR3 sequences in B-1a **(I)** and B-1b cells **(J)** from TET2-KO and WT mice. Each pair of bars represents the count of V-J associations combined from all samples. Blue and orange represent WT and TET2-KO mice, respectively. Sequences were pooled from mice from the same cell type and condition (n: B-1a: WT = 4, KO = 4, B-1b: WT = 4, KO = 3).

Circos plots (**Figures 7A–H**), measuring the relative frequency of each V-J pairing revealed a greater abundance of V_H_1-J _H_1, V_H_11-J _H_1, and V_H_12-J _H_4 in B-1a cells from TET2-KO mice compared to control (**Figure 7I**), suggesting that TET2 has an important role in specific V-J recombination of B-1a cells. These specific recombination events could be important for creating the over-representation of the specific CDR3s in B-1a cells from TET2-KO mice. The increase in V_H_12-J _H_4 pairing in TET2-KO mice was also seen in the B-1b cells but only constituted 5% of all pairings compared to over 20% in the B-1a cells (**Figures 7I–J**). These data are consistent with the loss of TET2 generating a more pronounced effect on the BCR in B-1a cells compared to B-1b cells.

## Discussion

Murine B cells can broadly be divided into B-2 cells, which are derived from BM precursors and include conventional follicular and marginal zone B cells, and B-1 cells, which are largely fetal liver-derived and persist in adults through self-renewal (60, 62–65). These B cell subtypes are developmentally, functionally, and phenotypically distinct (7, 18, 66–70). Given their self-renewal capacity, we hypothesized that B-1 cells may be regulated by TET2, an epigenetic modulator that has been implicated in the clonal expansion of hematopoietic cells leading to disorders such as myelodysplastic syndromes (MDS) (30–34) and acute myeloid leukemia (AML) (27–30). Indeed, the results of the present study identified an important role for TET2 in regulating B cell numbers in specific niches. However, further studies are needed to determine if this is an effect intrinsic to the loss of TET2 specifically in B cells. Even if these findings are secondary to TET2 loss in another cell type, they still have potential relevance to diseases regulated by IgMs produced by B-1 cells such as infection (71–77), atherosclerosis (78–84), and obesity-related metabolic dysfunction (85, 86). Several human genetic variants of TET2 with loss of function have been identified (87–89) and these could have a broad impact similar to global TET2 deletion in mice, resulting directly or indirectly in modulating the anti-inflammatory effects of IgM-producing B cells.

B-1 cells have been shown to have important roles in the first line of defense against pathogens (71–77) and in mediating a reduction of inflammation (8, 16, 60, 78, 85, 90). One of the major mechanisms mediating this effect is their production of IgM that can recognize pattern-associated molecular pattern (PAMPs) and danger-associated molecular patterns (DAMPs) such as phosphorylcholine on the cell wall of *Streptococcus pneumoniae* (73, 77, 91) and oxidation-specific epitopes (OSEs) on lipoproteins (92, 93). OSEs on lipoproteins and apoptotic cells can fuel disease-associated inflammation (93, 94) and IgM to these neoepitopes can inhibit their induction of inflammatory responses (95, 96). Our study presents novel findings that the global loss of TET2 increased B-1 cell number, circulating IgM level, and BCR specificity, all factors that could affect the immune response against PAMPs and DAMPs.

The first major phenotype we observed due to the global loss of TET2 was an elevation in the frequency and number of all B cell subtypes in the peritoneal cavity (**Figures 1G, J**). Yet, in the specialized niches that promote B cell effector function, such as antibody production, only the frequency of B-1a cells in the BM (**Figure 1H**), and B-1b frequency and number in the spleen (**Figures 1I, L**), were higher in the TET2-KO compared to WT mice. The mechanism responsible for these subset and niche-specific increases in cell number remains unclear and requires further study to determine if proliferation, increased cell survival, or migration are responsible. As B-1a cells self-renew like hematopoietic stem cells (HSCs) (15, 63, 97–100), and this self-renewal property is enhanced in HSCs with TET2-KO (24, 25, 30, 34, 101, 102), enhanced self-renewal of B-1a cells from TET2-KO animals may explain the increase in B-1a cells in the peritoneal cavity.

The genes and pathways that were different in B-1 cells from TET2-KO mice compared to control, particularly in the B-1a cells, were immunoglobulin-related and they were expressed at a lower level (**Figures 2C**, **3**). There was a predominance of kappa light chain genes that were less expressed, in addition to several V_H_ genes, leading us to hypothesize that loss of TET2 may be limiting the expression of certain variable region genes, which allows for specific antigen recognition of foreign or neo-antigens (15, 103–106). To further investigate those differences, we performed BCR analysis using our RNASeq data.

Historically, BCR identification from sequencing was facilitated by well-established algorithms like MiXCR (107) or BALDR (108) using V-D-J enriched or single-cell RNASeq data. However, the associated costs and impracticality of research studies focusing on low-frequency cell populations were limiting factors for broader application. The introduction of the TRUST4 algorithm by Song et al. (57) enabled the accurate detection of BCR and TCR repertoire from bulk RNASeq data. This innovation diminished the financial burden of data generation and allowed for the re-utilization of previously generated data, limiting redundancy and resources required for BCR/TCR analysis and providing opportunities for potential clinical applications. While the results are not at single-cell resolution, they offer valuable insight into the diversity of immune cell receptor repertoire and specificity. To date, a limited number of studies have performed an analogous analysis in bulk RNASeq data (109–111).

The constant region of the BCR determines the effector function of the antibody. There were no differences in the transcript expression levels of antibody isotypes IgG, IgD, and IgA (**Figure 4A**). However, there was a statistically significant increase in transcript expression of IgM, the main isotype produced by B-1 cells (10, 16, 90, 112), in the B-1a cells from TET2-KO compared to WT mice suggesting that TET2 may inhibit factors responsible for encoding the constant region downstream of the V region on chromosome 14 that determines antibody isotype or TET2 may limit isotype switching in B-1a cells (**Figure 4B**). These data are consistent with no changes observed in the circulating IgG level while there was an increase in circulating IgM in the TET2-KO mice compared to the control. We could not conclude if the increase in total IgM was due to increased IgM secretion on a per-cell basis or due to the increase in overall cell number (**Figure 4C**). However, increased IgM levels could also be due to the increase in B-1 numbers in the spleen and bone marrow. Additionally, there was an increase in MZB cell number in the spleens from TET2-KO mice compared to WT, another potential source of IgM from TET2-KO and WT mice (**Supplementary Figure 6**).

While much more has been documented about the role of the heavy chain variable region, specifically the CDR3 of the BCR, less is known about the purpose of the light chain regarding its role in binding antigens (59, 106, 113–118). A study by Lio et al. revealed that double knockout of TET2 and TET3 in the early B cell stage impaired rearrangement at the Igκ locus (23). Our findings support previous research by detecting the lower expression of many Igκ genes, and indeed, while not reaching significance, overall kappa immunoglobulin usage is reduced in B-1b cells, but surprisingly not B-1a cells from TET2-KO mice compared to WT (**Supplementary Figure 9E**). There was also a significant reduction in the number of unique CDR3 sequences in B-1a cells and a trending reduction in B-1b cells from TET2-KO mice compared to WT (**Figure 2**; **Supplementary Figure 8**). Consistent with the reduced variety of CDR3 sequences, there is a higher number of replicated sequences in the light chain observed in the B-1a cells from the TET2-KO mice compared to WT, and the effect was also observed in B-1b cells to a lesser extent (**Supplementary Figure 8**). These data suggest that the BCR repertoire in the light chains of B-1a cells is more sensitive to loss of TET2 than in B-1b cells.

B-1a cells from TET2-KO mice had significantly fewer unique CDR3 sequences with 72% of the total CDR3 sequences representing replicates, suggesting that loss of TET2 impacts the diversity of antigen specificity in B-1 cells, particularly B-1a cells (**Figure 5B**). The more marked lack of antigen diversity in the B-1a cells from TET2-KO mice is consistent with B-1a cells predominantly originating from the fetal liver and persisting through self-renewal, and a role for TET2 in promoting expansion of rapidly self-renewing cells. While our study isolated B cells from the global TET2-KO and WT mice, it must be considered that the effects of loss of TET2 in other cells, such as cytokine-secreting macrophages, could be playing a role in influencing the selection of the B cell repertoire. Additionally, the presence of IgM itself can influence the selection of the B cell repertoire (119). In an analysis of V_H_–D_H_–J_H_ gene regions of the heavy chain, our data suggest that the restricted associations of V_H_–D_H_–J_H_ gene regions in the B-1a cells from TET2-KO mice could be responsible for the reduction in the number of unique CDR3 sequences. A study by Wong et al. identified a pathway whereby B-1a cells can bypass the need for a pre-BCR and generate a mature, albeit somewhat self-reactive, BCR directly (120). The V_H_12/V_K_4 pairing is typical for binding phosphatidylcholine, a lipid present in many bacteria membranes, and while V_H_12 frequency of use is increased in B-1a cells from TET2-KO mice, V_K_4 frequency of use is lower in B-1a cells from TET2-KO mice compared to WT (**Supplementary Figure 8, 9**) (120). It should be noted that both V_H_11 and J_H_1 are associated with early fetal characteristics, which supports the potential enhancement of self-renewal that loss of TET2 regulation may foster (18, 65, 69, 115). Our CDR3 and VDJ association data from the heavy chain provide further evidence in addition to the light chain data that B-1a cells are more profoundly impacted by global loss of TET2 compared to B-1b cells. The reason for this remains to be determined but may be due to the expression of CD5 by B-1a cells, given that studies have shown many of the malignant B cell samples with loss of TET2 express CD5 (101, 121, 122), but this connection requires further study.

**Figure 8:**
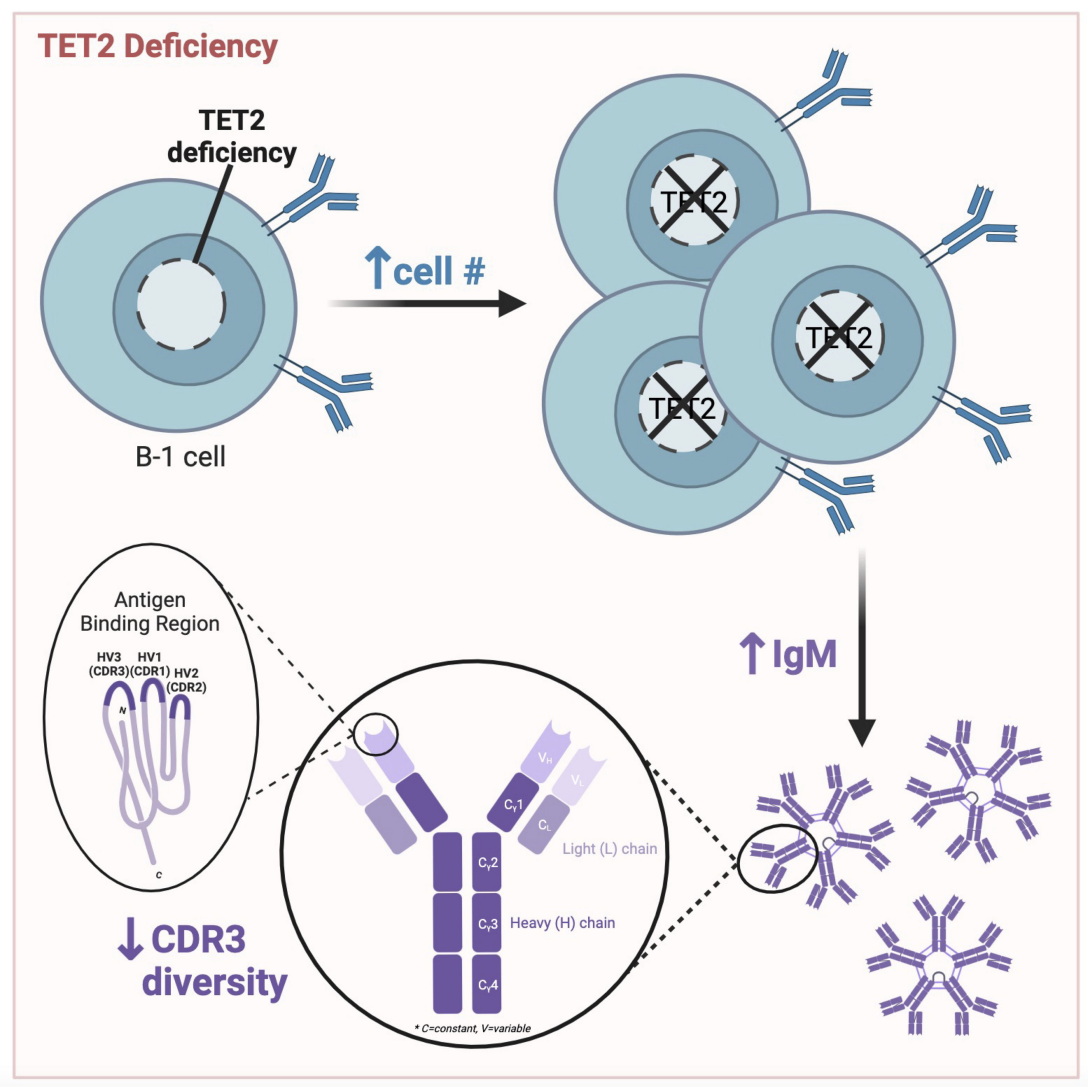
Graphical abstract of key findings. Peritoneal B cell number is increased, circulating IgM levels are elevated, and CDR3 sequence diversity is reduced in mice null for TET2 compared to WT mice. Figure made with BioRender.

Taken together, our data reveals that loss of TET2 influences IgM level and BCR repertoire, particularly in B-1a cells, which are key producers of natural IgM. Alteration to the antigen-specificity or abundance of B-1a-produced IgM may have consequences in the response to PAMPs and DAMPs and in regulating antigen-driven inflammation. Our data demonstrating that loss of TET2 increased B-1 cell subset numbers in antibody-producing niches and reduced CDR3 diversity suggests that TET2 may regulate the pool of antigen-specific IgM produced by B-1 cells (**Figure 8**) and underscores the need for further study of the impact and mechanisms whereby TET2 regulates B-1 cells, especially in the context of infection and diseases involving chronic inflammation.

## Data availability statement

The RNA sequencing data presented in the study is publicly available in GEO repository under GSE254998 accession (www.ncbi.nlm.nih.gov/geo/query/acc.cgi?acc=GSE254998).

## Ethics statement

The animal study was approved by UVA Animal Care and Use Committee. The study was conducted in accordance with the local legislation and institutional requirements.

## Author contributions

ED: Conceptualization, Formal Analysis, Funding acquisition, Investigation, Visualization, Writing – original draft, Writing – review & editing. MMu: Data curation, Formal Analysis, Funding acquisition, Investigation, Visualization, Writing – original draft, Writing – review & editing. CB: Investigation, Writing – review & editing. MMa: Investigation, Writing – review & editing. KR: Investigation, Writing – review & editing. TP: Writing – review & editing. JD: Writing – review & editing. LE: Writing – review & editing. CB: Writing – review & editing, Funding acquisition. SB: Supervision, Writing – review & editing. CM: Conceptualization, Funding acquisition, Supervision, Writing – review & editing.
