# Supplementary material for: Loss of TET2 increases B-1 cell number and IgM production while limiting CDR3 diversity
Source: Front Immunol. 2024 Mar 27;15:1380641. doi: 10.3389/fimmu.2024.1380641 (PMC11004297; doi:10.3389/fimmu.2024.1380641)
Supplement: Supplementary file 1 [file DataSheet_1.pdf]

Supplementary Material

Loss of TET2 increases B-1 cell number and IgM production while limiting CDR3 diversity

Emily Dennis[1,2,†], Maria Murach[1,3,†], Cassidy M.R. Blackburn[1], Melissa Marshall[1], Katherine Root[1], Tanyaporn Pattarabanjird[1], Justine Deroissart[4], Loren D. Erickson[1,2], Christoph J. Binder[4], Stefan Bekiranov[1,3], and Coleen A. McNamara[1,5,*]

[1]Beirne B. Carter Center for Immunology Research, University of Virginia, Charlottesville, VA, USA

[2]Department of Microbiology, Immunology, and Cancer Biology, University of Virginia, Charlottesville, VA, USA

[3]Department of Biochemistry and Molecular Genetics, University of Virginia, Charlottesville, VA, USA

[4]Department for Laboratory Medicine, Medical University of Vienna, Vienna, Austria

[5]Division of Cardiovascular Medicine, Department of Medicine, University of Virginia, Charlottesville, VA, USA

† These authors share first authorship

*** Correspondence:**
Coleen A. McNamara
cam8c@virginia.edu

Supplementary Figures

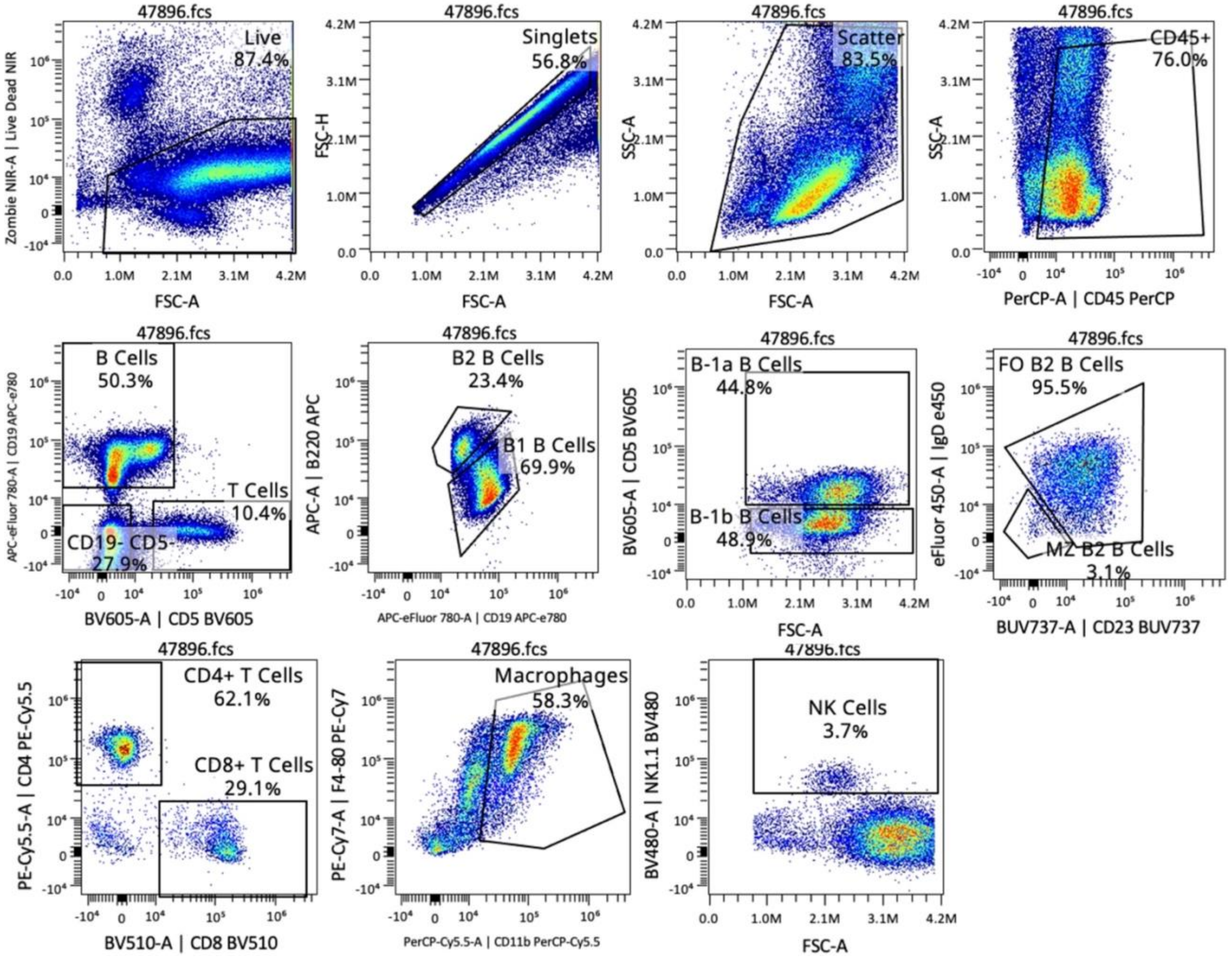

Supplemental Figure 1. Gating strategy for immunophenotyping the peritoneal cavity, bone marrow, and spleen of TET2-KO and WT mice. Representative plots are displayed from a peritoneal sample. B cells were defined as CD45+ CD19+; T cells were defined as CD45+ CD5+ CD19-; Macrophages (Macs) were defined as CD45+ CD5- CD19- F4-80+ CD11b+; and NK cells were defined as CD45+ CD5- CD19- NK1.1+.

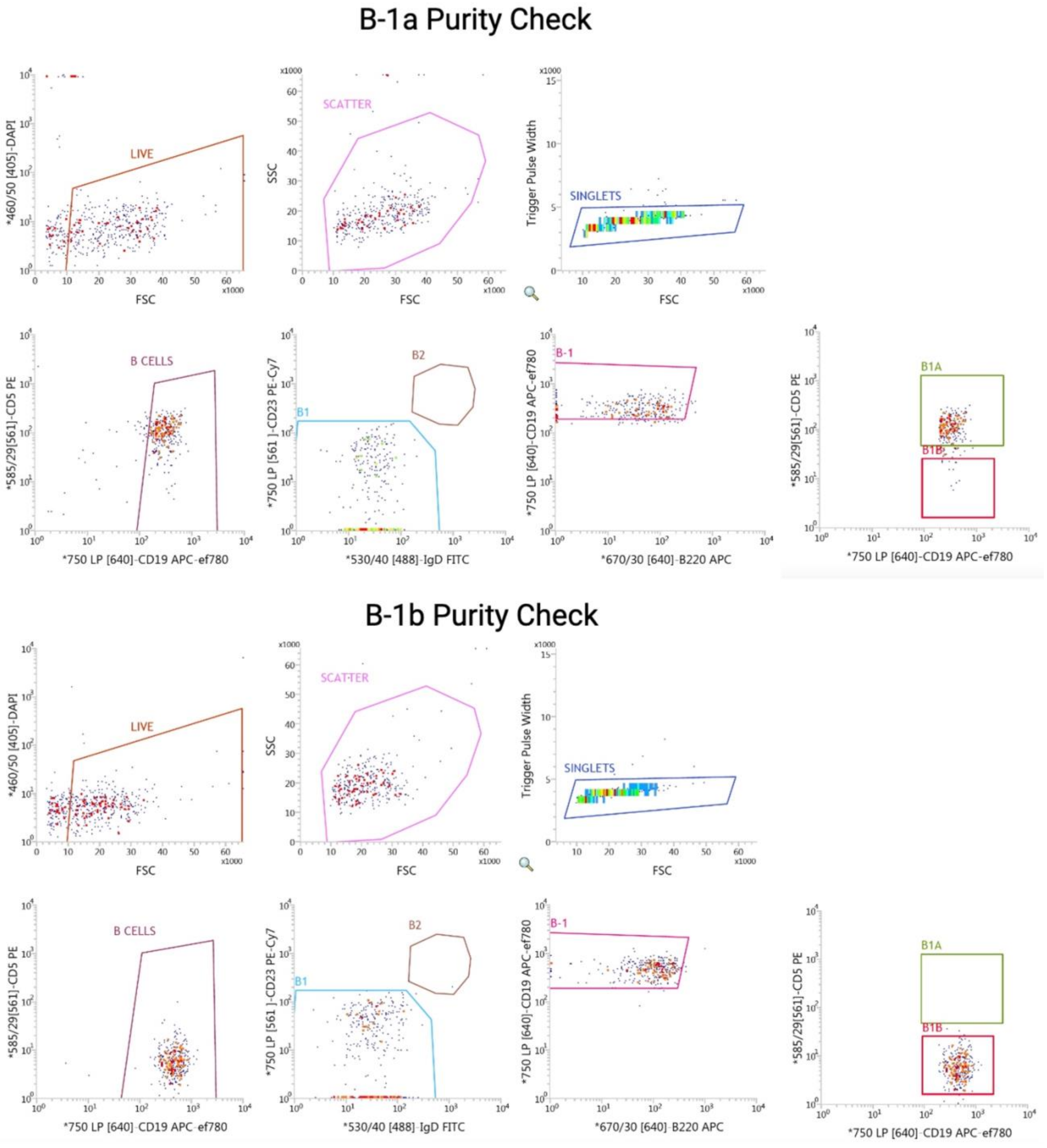

Supplemental Figure 2. Purity checks for B-1a (**top**) and B-1b cell (**bottom**) FAC-sort. We used the following gating strategy: live → scatter → singlets → total B cells → CD23lo IgDlo B-1 cells → B220lo B-1 cells → CD5+ B-1a cells or CD5- B-1b cells.

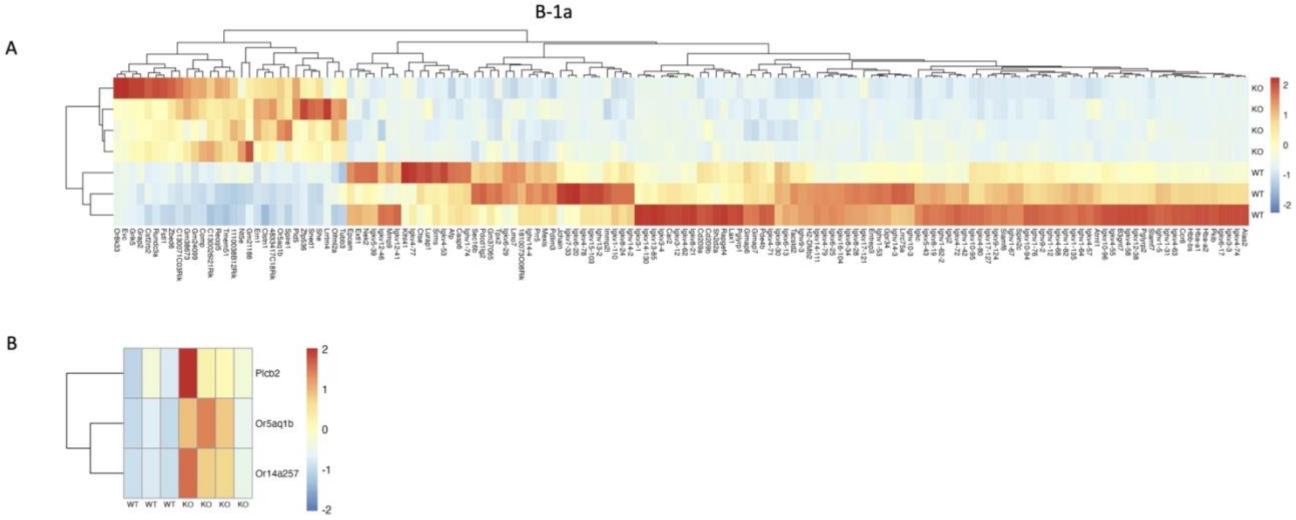

Supplemental Figure 3. Differentially expressed genes in B-1a cells from TET2-KO and WT mice. (**A**) Scaled expression of all differentially expressed in B-1a cells from TET2-KO and WT mice. Each column corresponds to a gene, and each row represents a sample from WT or TET2-KO. The expression was scaled for each gene (from -2 to 2) and is represented by red for high and blue for low expression values. (**B**) Scaled expression of differentially expressed genes related to sensory perception of smell/sensory perception of chemical stimulus/olfactory receptor activity pathways.

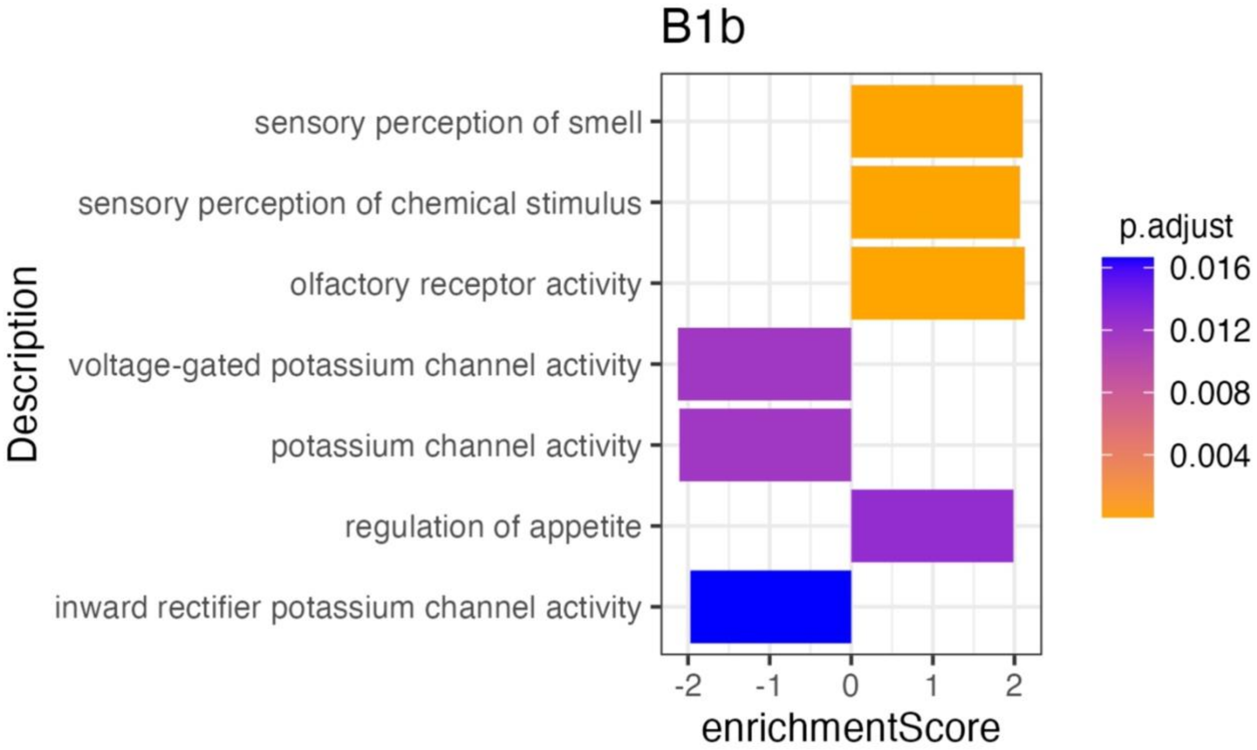

Supplemental Figure 4. Pathway analysis on differentially expressed genes in B-1b cells from TET2-KO and WT mice. Plot of enrichment scores from Gene Set Enrichment Analysis on differentially expressed genes in B-1b cells from TET2-KO and WT mice. The axis represents the enrichment score (ES). Higher scores indicate greater enrichment of the gene set at one end of the ranked list of genes. ES measures the degree to which a gene set is overrepresented at the extremes of the entire ranked list. Enrichment scores are colored based on FDR-adjusted p-values.

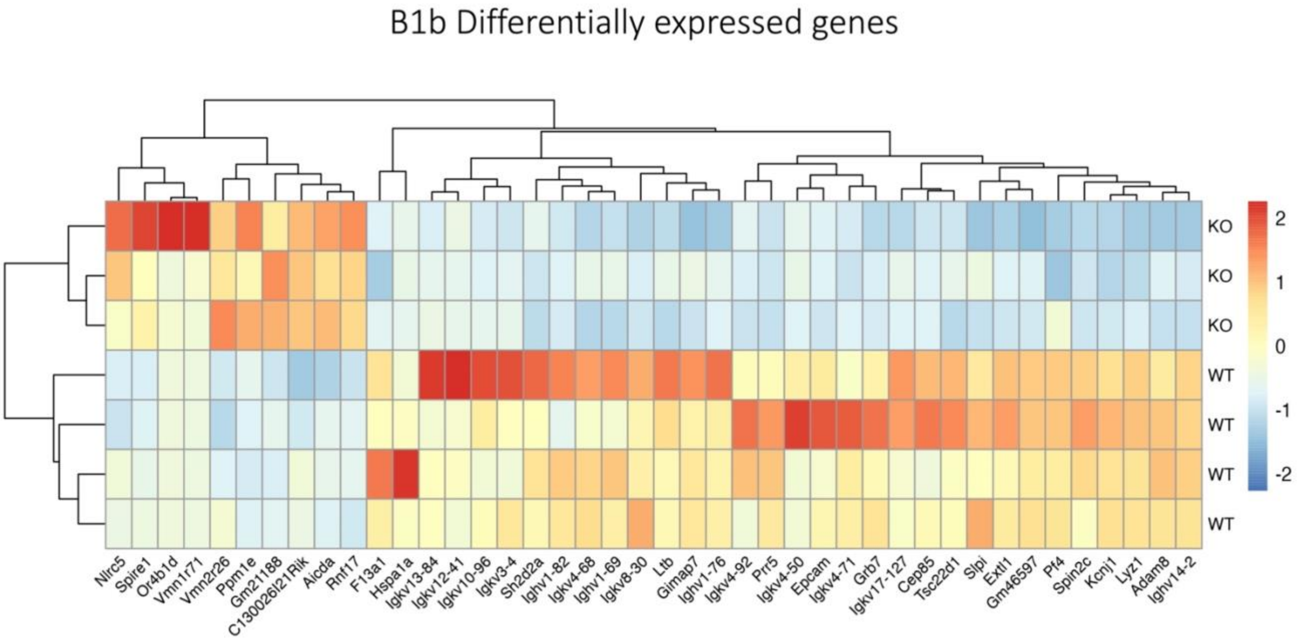

Supplemental Figure 5. Differentially expressed genes in B-1b cells from TET2-KO and WT mice. Scaled expression of differentially expressed in B-1b cells from TET2-KO and WT mice. Each column corresponds to a gene, and each row represents a sample from WT or TET2-KO. The expression was scaled for each gene (from -2 to 2) and is represented by red for high and blue for low expression values.

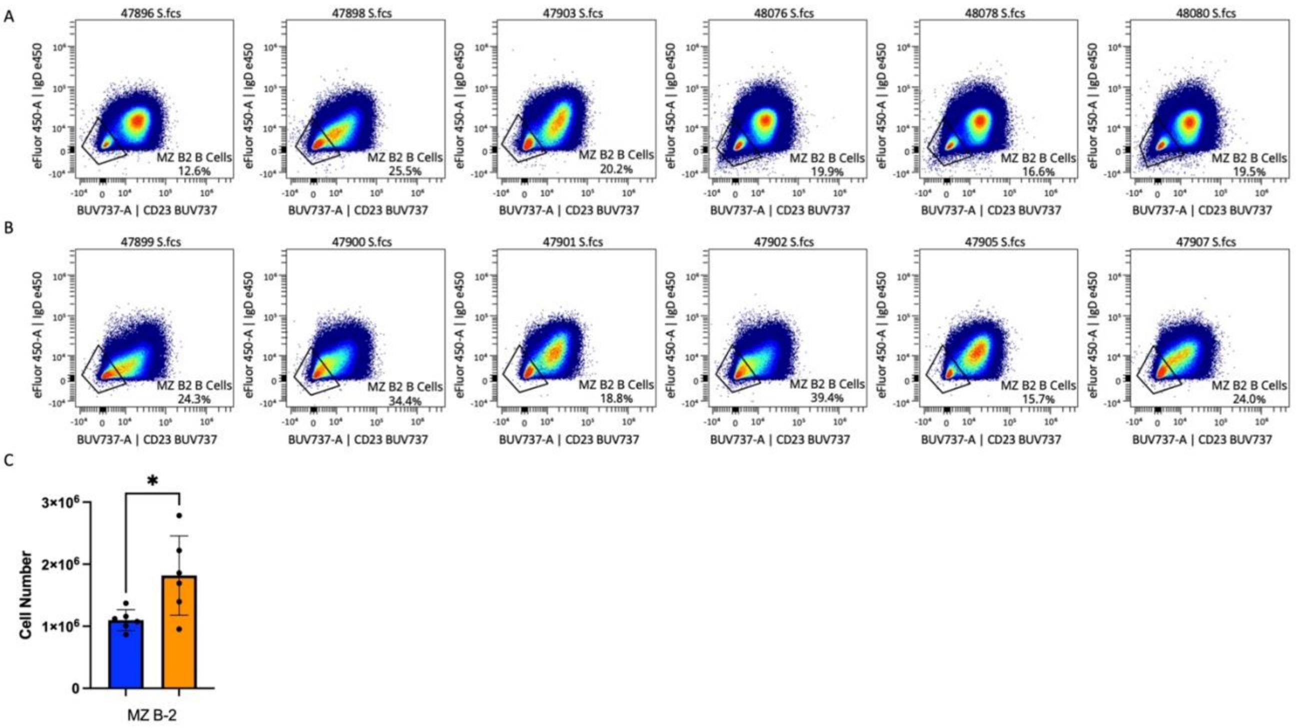

Supplemental Figure 6. Flow cytometry analysis of splenic Marginal Zone B-2 (MZB) cell population from TET2-KO and WT mice. (**A, B**) Density scatterplots display MZB cells from the spleens of TET2-KO (**B**) and WT mice (**A**). The gate percentage displayed on the plot is from the total B-2 cell population. (**C**) Bar chart displaying the total number of MZB cells from the spleens of TET2-KO and WT mice. Blue and orange represent WT (n = 6) and TET2-KO mice (n = 6), respectively. Statistical significance was assessed using the Mann Whitney Signed Rank Test between WT and KO conditions (* $p < 0.05$).

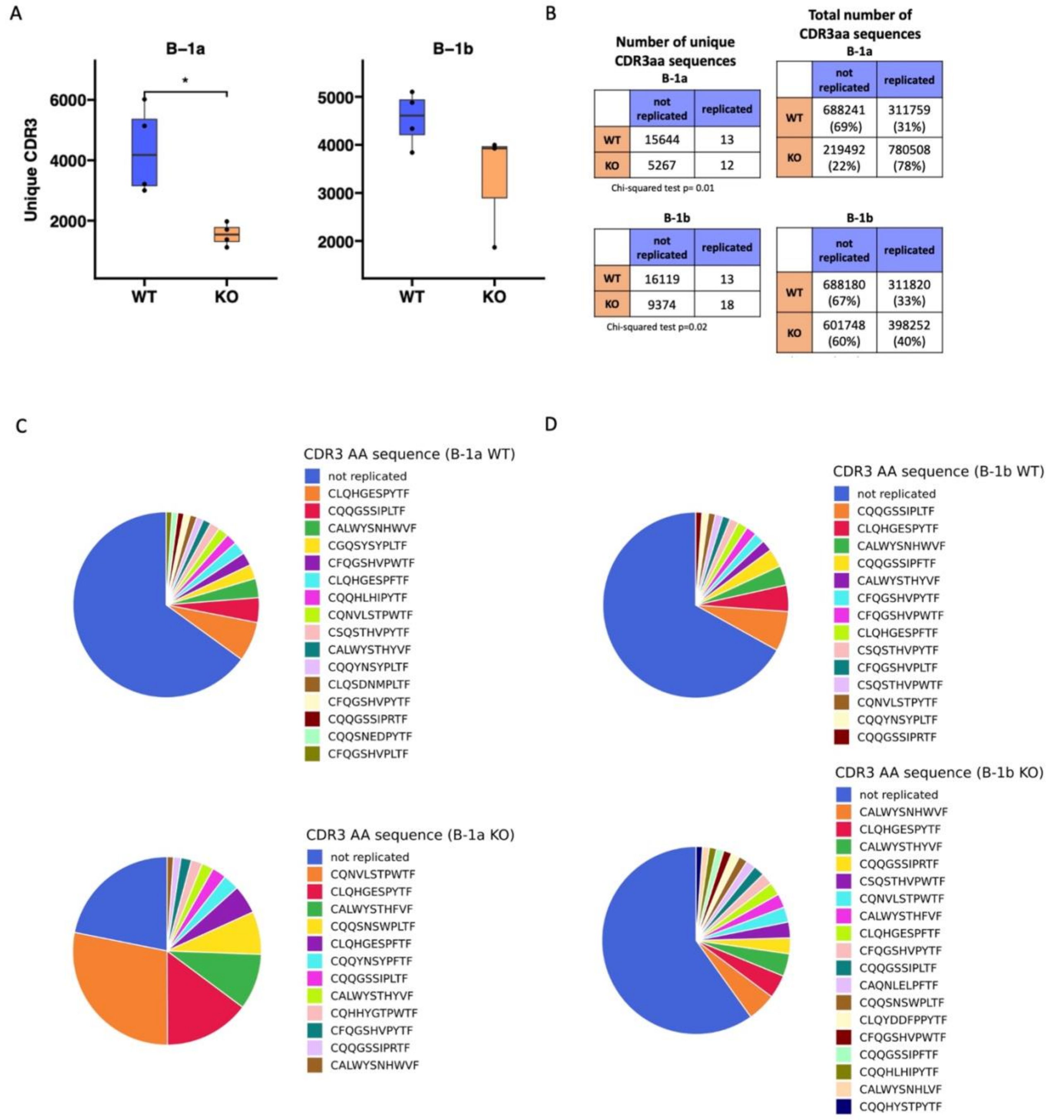

Supplemental Figure 7. Light chain CDR3 sequence analysis in B-1a and B-1b cells from TET2-KO and WT mice. (**A**) The number of unique CDR3 sequences identified by TRUST4 in B-1a and B-1b cells from TET2-KO and WT mice. (**B**) Contingency tables derived to assess the association between the number of unique CDR3 amino acid sequences (**left**) and total number of unique CDR3 amino acid sequences (**right**) with the mutant status of the mice (i.e., WT or TET2-KO) in B-1a (**top**) and B-1b cells (**bottom**). Chi-squared test was used to assess the significance of these associations. (**C, D**) Annotated pie charts depicting the proportion of CDR3 sequences that are unique and the sequence and proportion of the replicated sequences in B-1a (**C**) and B-1b (**D**) cells from WT (**top**) and TET2-KO mice (**bottom**). Statistical significance was assessed using the Mann Whitney Signed Rank Test between WT and KO conditions (* $p < 0.05$).

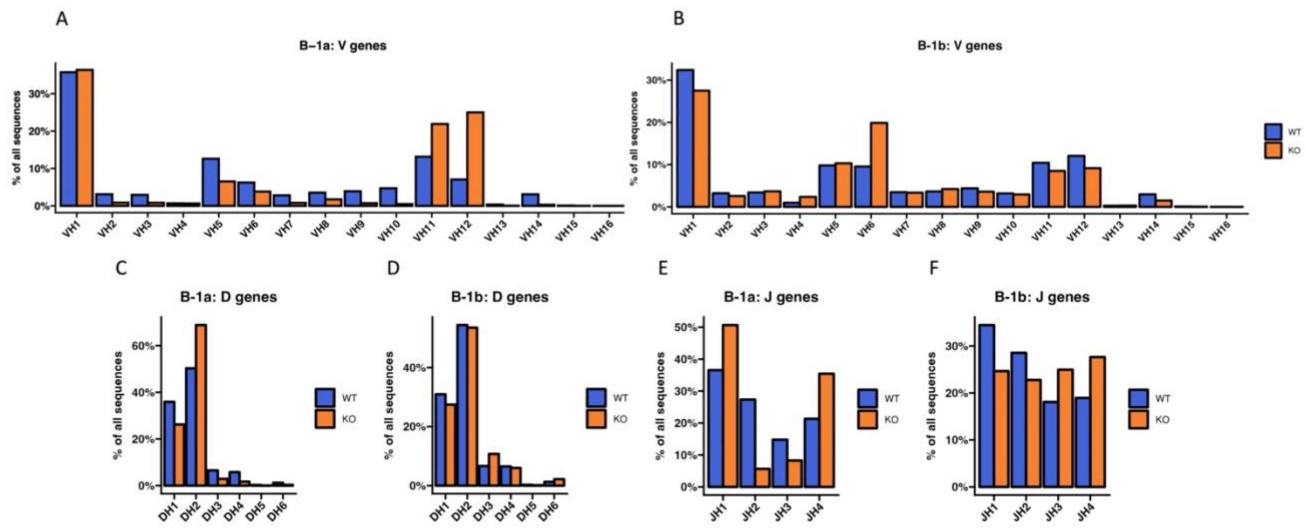

Supplemental Figure 8. V_H - D_H - J_H analysis of B-1a and B-1b cells from TET2-KO and WT mice. (**A-F**) The percentage of CDR3 sequences identified by TRUST4 utilizing V segments is displayed for B-1a (**A**) and B-1b cells (**B**), D segments for B-1a (**C**) and B-1b cells (**D**), and J segments for B-1a (**E**) and B-1b cells (**F**) from TET2-KO and WT mice. Each pair of bars represents the distribution of frequency values for a specific V, D, or J gene based on pooled gene frequencies from mice from the same cell type and condition. Blue and orange represent WT and TET2-KO mice, respectively.

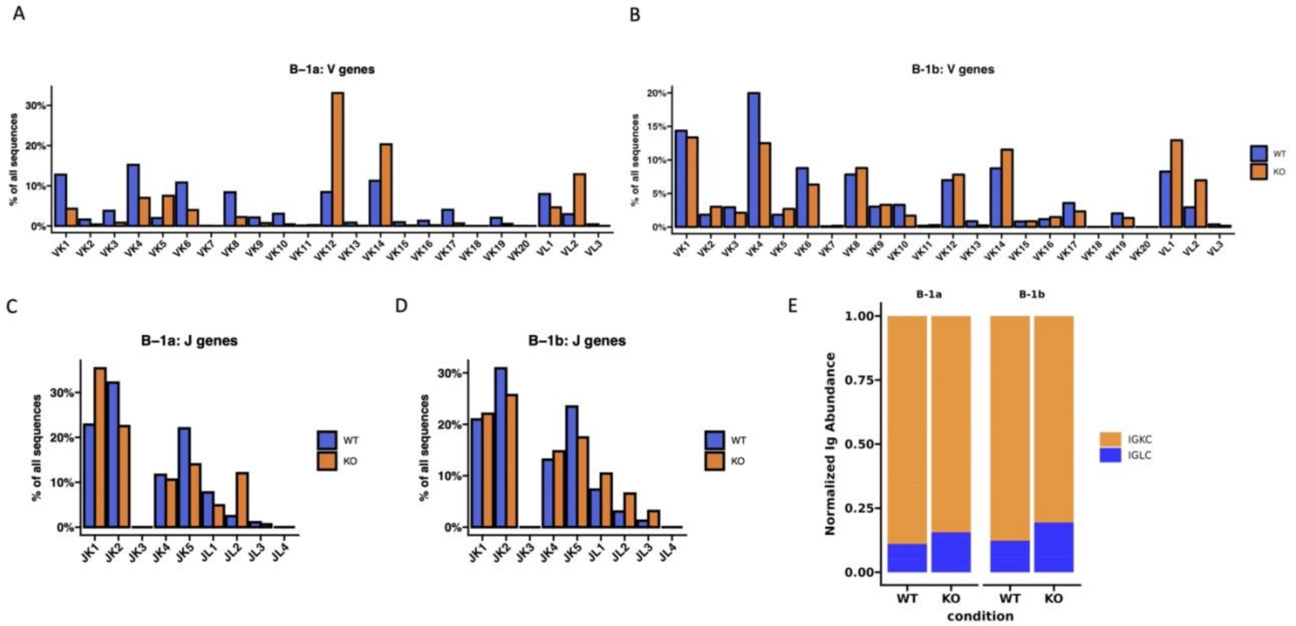

Supplemental Figure 9. $V_{K/L}$ – $J_{K/L}$ and kappa/lambda analysis of B-1a and B-1b cells from TET2-KO and WT mice. (**A-D**) The percentage of CDR3 sequences identified by TRUST4 utilizing V segments is displayed for B-1a (**A**) and B-1b cells (**B**) and J segments for B-1a (**C**) and B-1b cells (**D**) from TET2-KO and WT mice. (**E**) Bar chart showing the distribution of kappa and lambda usage identified by TRUST4 in B-1a and B-1b cells from TET2-KO and WT mice. Each pair of bars represents the distribution of frequency values for a specific V or J gene based on pooled gene frequencies from mice from the same cell type and condition. Blue and orange represent WT and TET2-KO mice, respectively.
